# Immunotherapy in Head and Neck Cancer—Where Are We Now and Where Are We Headed?

**DOI:** 10.3390/ijms27020987

**Published:** 2026-01-19

**Authors:** Rafał Becht, Kajetan Kiełbowski, Paulina Żukowska, Robert Kowalczyk, Sebastian Ochenduszko, Inmaculada Maestu Maiques, Katarzyna Radomska

**Affiliations:** 1 Department of Clinical Oncology, Chemotherapy and Cancer Immunotherapy, Pomeranian University of Medicine, 71-252 Szczecin, Poland; kajetan.kielbowski@onet.pl (K.K.); paulina.zukowska@pum.edu.pl (P.Ż.); 2 Maxillo-Facial Surgery Clinic, Pomeranian Medical University, 71-252 Szczecin, Poland; 3 Department of Oncology, Dr. Peset University Hospital, Av.de Gaspar Aguilar 90, 46017 Valencia, Spain; 4 Department of Otolaryngology, Pomeranian University of Medicine, 71-252 Szczecin, Poland; katarzyna.radomska@pum.edu.pl

**Keywords:** head and neck cancer, immunotherapy, systemic treatment, immune checkpoint inhibitors, biomarkers

## Abstract

Head and neck cancer (HNC) encompasses tumors located within the oral cavity, sinonasal cavity, pharynx, and larynx. It is the sixth most common cancer worldwide. Current treatment methods in HNC patients involve radical surgery, radical radiotherapy, and concomitant chemoradiotherapy, along with adjuvant and induction therapies. Accumulating trials examine the role of immunotherapy in patients with HNC. The results of the CheckMate-141 and KEYNOTE-048 trials demonstrated the benefits of using immunotherapy in patients with metastatic or recurrent HNC. Subsequently, numerous other immunotherapy-based protocols have been evaluated. Then, KEYNOTE-689 successfully implemented immunotherapy in patients with locally advanced disease. This review aims to comprehensively present the landscape of immunotherapy opportunities in patients with HNC. It summarizes completed key clinical trials that led to the approval of immunotherapy in HNC and presents currently performed trials with highly expected results. Furthermore, it discusses methods to improve immunotherapy outcomes in the cohort of HNC patients, describes the current role of immunotherapy in HNC, and presents future perspectives of this type of treatment.

## 1. Introduction

**Figure 1:**
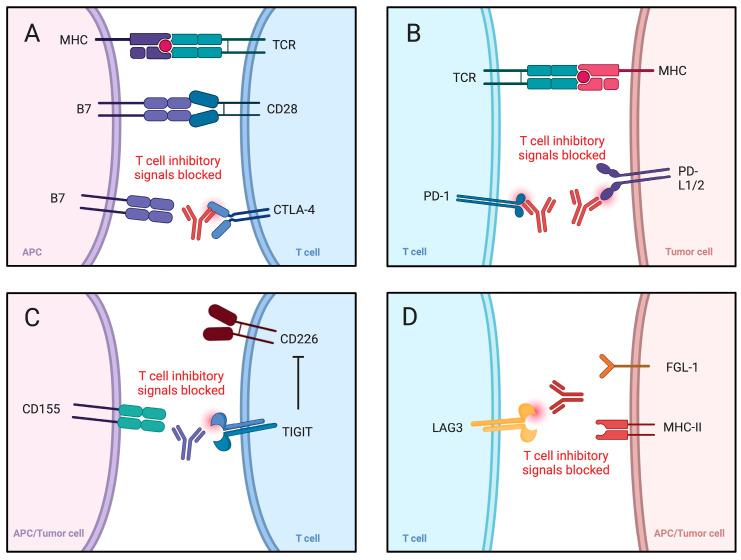
Immune checkpoint inhibitors. (**A**,**B**) present classic and widely known approaches targeting PD-1/PD-L1 axis and CTLA-4. (**C**,**D**) depict still explored areas targeting TIGIT and LAG3. Created in BioRender. Kiełbowski, K. (2026) https://BioRender.com/tnilx03.

Immunotherapy has entered clinical practice and became one of the pillars of systemic oncological treatment. It has been registered in a number of indications, including gastric cancer, urothelial cancer, melanoma, and urothelial carcinoma, among others. Despite the already known benefits of immunotherapy, accumulating studies and clinical trials continue to explore its novel properties and potential clinical implementation. The vast array of already approved immunotherapeutics include drugs such as pembrolizumab, nivolumab, ipilimumab, durvalumab, tremelimumab, dostarlimab, cemiplimab, tislelizumab, and atezolizumab. These agents belong to the family known as immune checkpoint inhibitors (ICIs) (Table 1). They interact with molecules such as programmed cell death protein 1, its ligand (PD-1/PD-L1), and cytotoxic T-lymphocyte-associated protein 4 (CTLA-4), thus restoring cytotoxic properties of T cells (Figure 1).

Head and neck cancer (HNC) is a term used to describe cancers located within the oral cavity, sinonasal cavity, pharynx, and larynx [12]. According to the GLOBOCAN 2022 estimations, HNC is the sixth most prevalent cancer, with current predictions suggesting a continuing rise in incidence [13,14]. Histologically, HNC primarily involves squamous cell carcinoma, having its origins in the epithelial lining of the involved anatomical regions. Among the risk factors for developing HNC, we can distinguish tobacco use, alcohol consumption, diets rich in processed meat, and HPV infection, among others [12,14].

**Figure 2:**
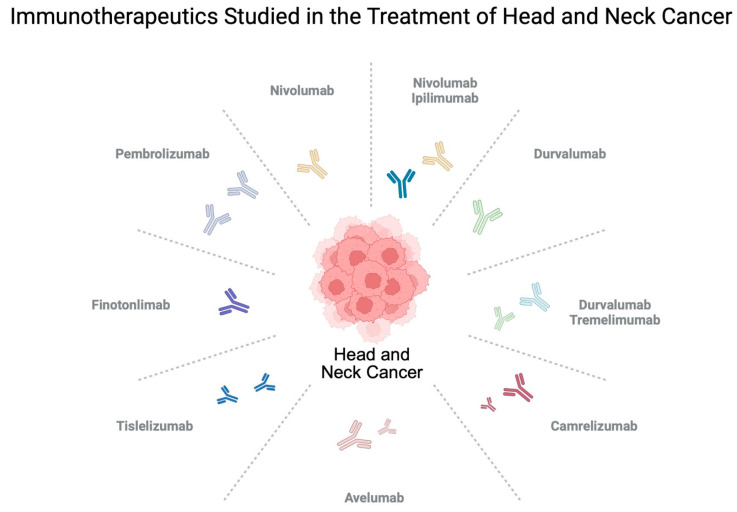
A variety immunotherapies is being studied in patients with head and neck cancer. Created in BioRender. Kiełbowski, K. (2026) https://BioRender.com/yyt80ph.

Treatment guidelines differ depending on the location and stage of the disease. In a localized stage, radical surgery followed by radiotherapy is frequently recommended. Radical radiotherapy is considered as an option for patients with inoperable tumors. With more advanced disease, patients can be offered concomitant chemoradiotherapy. Immunotherapy is currently reserved for patients with metastatic or recurrent disease in which curative radiotherapy or surgery are not possible [15]. The area of immunotherapy in HNC is consistently increasing, with accumulating clinical trials being performed to evaluate its potential benefits in this indication (Figure 2). This treatment method has begun to be recommended for patients with locally advanced disease as well. The aim of the present article is to comprehensively summarize current knowledge regarding the use of immunotherapy in patients with HNC. A key role of this review is to present successful clinical trials and compare efficacy between different regiments. Furthermore, current evidence regarding the potential future of using immunotherapy in HNC patients is depicted. Additionally, modern research methods are presented to demonstrate their clinical translation potential and probable benefits in patients treated with immunotherapy.

## 2. Review Methodology

The present article is a narrative review that aims to present the most recent landscape of immunotherapy use in patients with HNC. A comprehensive literature search through the PubMed database was performed. The following keywords and their combinations were used: ‘head and neck cancer’, ‘locally advanced’, ‘metastatic or recurrent’, ‘immunotherapy’, ‘clinical trial’, and ‘immune checkpoint inhibitor’. Only articles written in English were considered. Priority was given to studies published between 2025 and 2020 and to phase III clinical trials. Moreover, clinicaltrials.gov and conference abstracts from ESMO and ASCO meetings were analyzed. Real-world data and retrospective studies were analyzed as well. No formal risk-of-bias assessment was conducted.

## 3. Immunotherapy in Head and Neck Cancer

### 3.1. Locally Advanced Head and Neck Cancer

This section discusses locally advanced disease stage III/IV p16-negative oropharyngeal cancer, larynx and hypopharynx cancer, and T3-4/N0-3 and T0-4/N1-3 p16-positive oropharyngeal cancer [15]. The 2020 ESMO guidelines recommend surgery with adjuvant therapy or concomitant chemo-radiotherapy. Chemotherapy usually involves a platinum-based drug, which can be combined with 5-fluorouracil in patients that are unfit for high-dose cisplatin administration [15]. Accumulating trials examine the potential use of immunotherapy in different approaches in patients with locally advanced disease. For instance, immunotherapy can accompany concurrent chemoradiotherapy or radical radiotherapy. Furthermore, immunotherapeutics are examined in neoadjuvant settings or with induction chemotherapy.

Firstly, several trials have studied immunotherapeutics with concomitant chemoradiotherapy or radiotherapy. KEYNOTE-412 analyzed the addition of pembrolizumab to chemoradiotherapy in patients with locally advanced HNC. A cohort treated with immunotherapy received pembrolizumab before, during, and after chemoradiotherapy, with a total of 17 cycles. Nevertheless, the use of immunotherapy was not associated with significantly better event-free survival compared to the control group [16]. In another study performed by Johnson et al. [17], the authors examined a combination of nivolumab and ipilimumab given prior to, during, and after radiotherapy. It was a small sample trial, with 24 patients enrolled. However, it was associated with highly promising responses, including 79% of patients achieving PR and 21% of patients with CR. Furthermore, median progression-free survival (PFS) in the cohort was not reached, while the 1-, 2-, and 3-year locoregional control rates were 100%, 100%, and 95%, respectively. However, in the GORTEC 2015-01 PembroRad phase II trial, the authors did not observe significant benefits when combining pembrolizumab with radiotherapy compared to a cetuximab–radiotherapy combination in terms of locoregional control [18]. The results of these trials warrant further research into concomitant double immunoradiotherapy to achieve satisfactory regional control.

Secondly, efficacy and safety of immunotherapy were assessed in the neoadjuvant setting, in which treatment is administered before radical local therapy. The aim of this approach is to decrease the size of the treated lesion and to increase chances of positive outcomes of radical therapy. Currently, there is no consensus on the proper approach. Moreover, different strategies are investigated if patients are considered resectable or not. Firstly, when comparing chemotherapy and immunotherapy in patients with surgically available disease, Pedroso et al. [19] conducted a meta-analysis that investigated the effects of chemotherapy vs. immunotherapy. Pooled analyses revealed that the nivolumab–ipilumumab regimen was associated with the greatest rate of pathological PR status, but also with the highest incident rate of surgical complications.

Wu and colleagues [20] examined 48 patients that underwent neoadjuvant treatment with camrelizumab, cisplatin, and nab-paclitaxel. After the three cycles of treatment, patients underwent either surgical or non-surgical treatment. The authors noted highly positive response rates, with an overall response rate (ORR) of 89.6%. Among responding patients, CR and PR were achieved by 10 and 33 patients, respectively. Among resected patients, pathological CR or major pathological response was diagnosed in 17 cases. Researchers also studied potential markers of response. They observed better radiographic response in HPV-positive patients and in those who had higher density of CD8+ T cells and macrophages within the tumor. On the contrary, lower radiological responses were seen in harborers of TP53 and TERT abnormalities. The IMCISION trial evaluated the use of nivolumab with ipilimumab in patients with resectable T2-T4, N0-N3b, and M0 HNC. Thirty-five percent of patients achieved major pathological response after two courses of immunotherapy [21]. In recently published results of the phase II NeoRTPC02 trial, resectable locally advanced HNC patients received neoadjuvant treatment composed of tislelizumab, albumin-bound paclitaxel, and low-dose radiotherapy. None of the 28 included patients were classified with PD. By contrast, 7.1% and 57.1% of patients achieved CR and PR, respectively. The rest of the patients had SD. The neoadjuvant therapy was composed of two cycles, and 35.7% of patients experienced grade 3–4 AEs, with neutropenia and leucopenia being the most common grade 3 events. Biomarker analysis demonstrated that greater pretreatment expression of CD20 correlated with pathological response [22]. One of the most influential clinical trials in patients with locally advanced HNC is the KEYNOTE-689 phase III trial, which investigated the use of pembrolizumab in neoadjuvant and adjuvant settings. In this trial, the group treated with pembrolizumab was compared to the cohort that received adjuvant radiotherapy with or without cisplatin after surgical treatment. Importantly, the results showed that pembrolizumab significantly increased event-free survival rate [23], which is already demonstrated in the latest NCCN treatment guidelines. The results of another phase III trial, named NIVOPOSTOP, are still expected. The trial randomized patients postoperatively to receive radiochemotherapy alone or with nivolumab. According to the information presented at the 2025 ASCO Meeting, the immunotherapy combination achieved higher disease-free survival (63.1% vs. 52.5%) [24]. In addition, promising results of an interesting study were presented at the ESMO 2025 Congress. The CAMORAL trial evaluated the use of neoadjuvant camrelizumab with chemotherapy together with adjuvant immunotherapy in patients with locally advanced HNC. In the study group, 2-year event-free survival and OS were 77.8% and 92%, respectively. These results were significantly greater than in the control group (46.1% and 69.1%, respectively). Furthermore, the investigated combination showed better rates of major pathological response and pathological CR (47.6% vs. 25.4%) [25].

**Figure 3:**
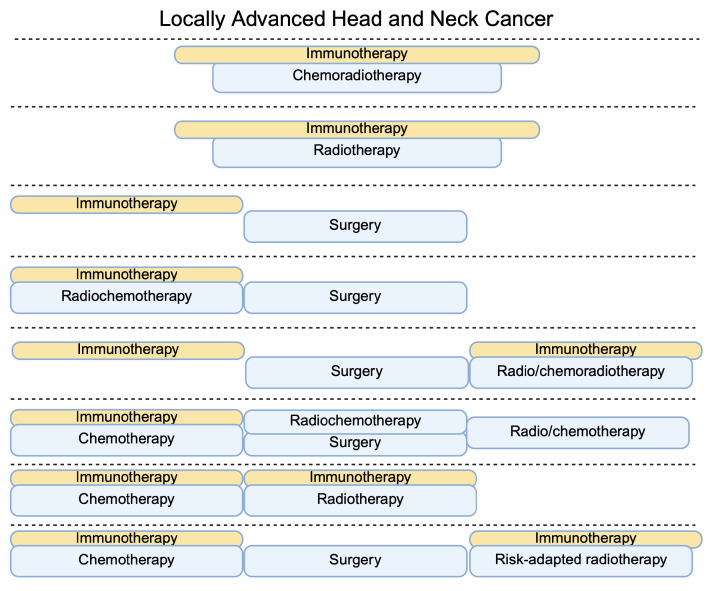
Selected treatment strategies examined in locally advanced head and neck cancer involving immunotherapy. Created in BioRender. Kiełbowski, K. (2026) https://BioRender.com/ee9wd4m.

Apart from neoadjuvant strategies in resectable patients, several studies investigated the addition of immunotherapy to induction chemotherapy. Li et al. [26] studied the combination of sintilimab with chemotherapy in patients later treated with surgery or definite radiochemotherapy, also followed by adjuvant treatment. Significantly more patients achieved overall response rate (ORR) in the cohort administered with immunotherapy. Moreover, the study group also demonstrated significantly better PFS. In a more recent single-arm study by Georgy et al. [27], researchers used low-dose nivolumab combined with induction chemotherapy (mainly regimens composed of platinum and taxane agents). Importantly, the results showed a highly promising efficacy, with 5.6% and 69.7% of patients achieving CR and PR, respectively. Moreover, 31.6% of patients underwent surgical therapy. An interesting approach was demonstrated in the CheckRad-CD8 trial, which evaluated the addition of immunotherapy (durvalumab + tremelimumab) to induction therapy. At the post-treatment rebiopsy, patients with increased abundance of CD8+ cells or with pathological CR were qualified for subsequent radioimmunotherapy. The majority of participants (76%) were subsequently treated with radioimmunotherapy. Two-year PFS and OS in this group were 72% and 84%, respectively. These values were much higher than in the group not qualified for radioimmunotherapy [28]. Figure 3 depicts an overview of the treatment strategies investigated in clinical trials of locally advanced head and neck cancer. The CompARE trial is a novel study that was presented at the ESMO Congress 2025. It evaluated the efficacy of neoadjuvant and adjuvant durvalumab. Even though the strategy is described as an induction therapy, the abstract does not provide information about the rate of surgical treatment. Currently, available results do not find benefits of the investigated approach [29]. The above-mentioned landscape of clinical trials in locally advanced disease demonstrates several critical points. Firstly, resectability status seems to play an important role. The KEYNOTE-412 trial included patients with unresected disease receiving concurrent immuno-chemo-radiotherapy. In contrast, the KEYNOTE-689 trial analyzed perioperative immunotherapy combined with surgery and adjuvant radiotherapy with or without cisplatin. The feasibility for local radical treatment could explain different results obtained in these studies. A similar situation could also explain the results of the IMvoke010 trial, which evaluated atezolizumab in HNC patients after multimodal definitive treatment. Immunotherapy did not improve clinical outcomes. However, over 60% of the included population did not receive surgical treatment as part of the definitive treatment [30]. Nevertheless, it needs to be acknowledged that the study group was heterogenous and the failed effects of atezolizumab are likely multifactorial. Secondly, the timing of immunotherapy is important. The benefits of neoadjuvant immunotherapies, depicted in the KEYNOTE-689 and CAMORAL trials, suggest the potential role of immunological priming in enhancing clinical outcomes. The use of concurrent immunotherapy (KEYNOTE-412) does not have immunological influence on ICIs. Additionally, neoadjuvant and adjuvant immunotherapies have distinct effects on the tumor microenvironment (TME). Specifically, neoadjuvant therapy can activate residing lymphocytes within the tumor. By contrast, adjuvant immunotherapy does not induce these effects, thus diminishing the beneficial role of ICIs on immune cells. It also needs to be acknowledged that neoadjuvant immunotherapy requires strong multidisciplinary cooperation. Progression while being treated with neoadjuvant immunotherapy can occur, which requires careful monitoring of patient condition and sooner initiation of radical treatment.

Furthermore, the role of individual drugs can also have effects. Perhaps this issue translates into low activity of durvalumab in the CompARE trial. The next aspect is the power of results. Smaller studies investigating concurrent chemoimmunotherapy in induction settings demonstrated clinical activity. However, phase III clinical trials are known for their rigorous criteria and analysis. For instance, the use of pembrolizumab in the KEYNOTE-412 trial did show clinical activity but it was not strong enough to meet the primary objective of event-free survival (log-rank *p* = 0.043; significance threshold *p* ≤ 0.024). Another variable is the HPV status. We now know that there can be p16/HPV discordance which affects clinical outcomes [31]. Taking into consideration the impact of HPV status on immunological function, its presence influences immunotherapy response. Presumably, the different compositions of HPV status in the studied population can affect the outcomes of clinical trials, which need to be acknowledged. The design of novel clinical trials must include information or stratify patients based on p16/HPV presence.

### 3.2. Recurrent/Metastatic Head and Neck Cancer

Before the introduction of immunotherapy, a first-line treatment option for HNC patients with recurrent/metastatic disease was the EXTREME protocol, which is composed of a platinum-based drug, 5-fluorouracil, and cetuximab, a targeted anti-EGFR monoclonal antibody. The standard of care was changed due to the results of the KEYNOTE-048 clinical trial, which demonstrated that pembrolizumab in monotherapy or in combination achieved better outcomes compared to the group treated with cetuximab with chemotherapy [32]. Recently, updated results confirmed the long-term benefits of immunotherapy. Specifically, 5-year survival rates were 14.4% for pembrolizumab in monotherapy compared to 6.5% in the EXTREME protocol [33]. Since both approaches (monotherapy and chemoimmunotherapy) demonstrated benefits over chemotherapy, the decision of which protocol to implement should be based on the performance status and predicted tolerability to the toxicities associated with chemotherapy. However, a recent meta-analysis performed by Meenu et al. [34] should be acknowledged. The authors studied the efficacy and safety of pembrolizumab alone or combined with chemotherapy. A total of 1006 and 448 patients were included in the monotherapy and combination groups, respectively. The ORR and adverse events favored pembrolizumab, while the odds ratio was not significant for OS and PFS. Despite the benefits of a single immunotherapy, the authors warrant potential biases that can limit driving clinical decisions [34]. Different approaches could be offered to patients with a low CPS score. In a subgroup analysis of the KEYNOTE-048 trial, patients with CPS < 1 demonstrated poor outcomes with pembrolizumab monotherapy [35]. The decision whether to introduce pembrolizumab monotherapy or a combination should also depend on the dynamics of progression. In patients with rapid progression and significant symptoms of the disease (such as organ function crisis or airway obturation), a combination of immunotherapy with chemotherapy should be considered. This approach provides the opportunity for a rapid improvement of clinical condition.

In an analysis by Lee et al. [36], the authors described the outcomes of 2577 patients with recurrent or metastatic HNC treated with pembrolizumab- or nivolumab-based protocols in first-line settings. Immunotherapy in monotherapy achieved significantly better overall survival (OS) compared to the EXTREME protocol (median survival 14.6 vs. 12.6 months). The combination of immunotherapy with chemotherapy only demonstrated a positive trend in survival. Interestingly, when patients were stratified according to their HPV status, significant effects on survival were observed only in the HPV-negative cohort.

Immunotherapy in patients with recurrent/metastatic HNC in first-line settings was recently investigated in another protocol. KEYNOTE-B10 is a single-arm phase IV clinical trial that investigated the use of pembrolizumab in combination with carboplatin and paclitaxel (CPXL combination). Researchers studied treatment effects in 101 patients and observed ORR in 49% of participants. Median OS and PFS were 13.1 and 5.6 months, respectively [37]. The promising anticancer activity suggested that the novel protocol could eliminate the drawbacks of 5-fluorouracil, including its long-term infusion and adverse events (AEs). Nevertheless, there is no comparative study evaluating the benefits of a pembrolizumab-CPXL combination compared to the pembrolizumab-mono or pembrolizumab-platinum-5-FU regimen. However, results of an indirect comparison were recently published. The network meta-analysis demonstrated that in terms of ORR, pembrolizumab-CPXL was similar to the EXTREME regimen, while it showed better results than the combination of pembrolizumab-platinum-5FU. In survival analysis, the protocol used in KEYNOTE-B10 was similar to the one used in KEYNOTE-048 and was better than EXTREME from the 12th month [38].

Recently, the efficacy of a combination of pembrolizumab and epacadostat, an indoleamine 2,3 dioxygenase 1 (IDO1), was described in the results of the KEYNOTE-669/ECHO-304 trial. In this study, patients previously untreated for recurrent/metastatic HNC were assigned to the groups of pembrolizumab monotherapy, pembrolizumab with epacadostat, or the EXTREME regimen. The primary endpoint of the study was the ORR, with results being similar in the pembrolizumab/epacadostat and the EXTREME cohorts (31% vs. 34%, respectively) [39]. Importantly, small samples (*n* = 34, *n* = 19, *n* = 34) are a limitation of the study, which could influence the results.

The KESTREL trial studied the potential role of durvalumab alone or in combination with tremelimumab in patients with recurrent or metastatic HNC. No significant differences were observed between the immunotherapy protocols and EXTREME regimen in terms of OS. However, in those patients that achieved partial response to immunotherapy, a significant survival benefit was noted. Specifically, median OS was three times longer in those patients as compared to the group that received the EXTREME protocol [40]. Blood tumor mutational burden was recently suggested to be a predictive factor for response to the combination of durvalumab and tremelimumab [41]. Combination therapy involving nivolumab with ipilimumab was non-superior to the EXTREME protocol as well [42].

**Figure 4:**
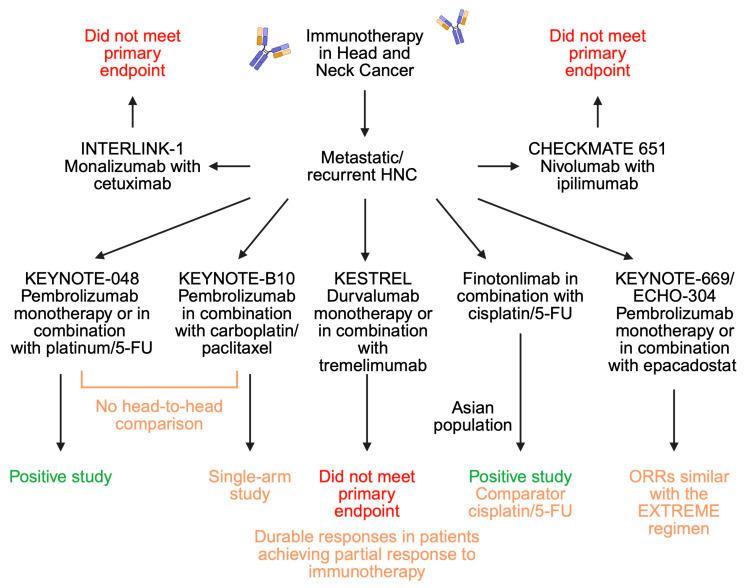
Overview of clinical trials examining the role of immunotherapy in metastatic head and neck cancer. Created in BioRender. Kiełbowski, K. (2026) https://BioRender.com/40q10x6.

A different approach was studied in the clinical trial performed by Shi and colleagues [43]. Researchers evaluated the use of finotonlimab (anti-PD-1 monoclonal antibody) combined with cisplatin and 5-fluorouracil in mainland Chinese patients. The control group received placebo with cisplatin and 5-fluorouracil. Survival was significantly greater in the study group, with median OS of 14.1 months in the study group and 10.5 in the control group. Median PFS was marginally greater in the finotonlimab group (*p* = 0.0493). The trial thus demonstrated benefits of immunotherapy to classic chemotherapy drugs. Unfortunately, the control group did not receive the EXTREME protocol, which makes it more difficult to compare these results to the KEYNOTE-048 trial. In the cohort of platinum-resistant or refractory recurrent/metastatic HNC, Merlano et al. [44] studied the use of avelumab (anti-PD-L1) in combination with cyclophosphamide and radiotherapy. The protocol was examined in 20 patients, in which 2, 3, and 7 patients achieved CR, PR, and SD, respectively. Median OF and PFS were 9.2 and 3 months, respectively. Recently, Fayette et al. [45] demonstrated the results of an interesting trial investigating the potential of immunotherapy targeting T cells and NK cells. Monalizumab is a monoclonal antibody that works as an immune checkpoint inhibitor by targeting CD94/NKG2A receptors [46]. In INTERLINK-1, a phase III clinical trial, the authors evaluated the combination of monalizumab with cetuximab in patients with recurrent/metastatic HNC. Despite a relatively novel and interesting approach, the suggested combination did not achieve better survival rates as compared to the cohort that received cetuximab with placebo [45]. Figure 4 summarizes the performed clinical trials that evaluated immunotherapy in patients with recurrent/metastatic HNC.

Choosing an appropriate second-line treatment option is still a challenge. With the relatively novel role of immunotherapy in recurrent/metastatic HNC, clinical trials investigating the potential role of immunotherapy switching or immunotherapy used after failure of prior ICIs is required. Such an approach is recommended in selected cases of melanoma [47]. In HNC, the CheckMate-141 trial confirmed the efficacy of nivolumab in patients that progressed on platinum-based chemotherapy [48]. A Delphi consensus statement supports the use of cetuximab with taxanes after progression on chemoimmunotherapy. If immunotherapy is used in monotherapy in the first-line treatment, cetuximab should be combined with chemotherapy; 100% of responders agreed that immunotherapy should be used when patients progress on cetuximab with chemotherapy. The latter statement involves patients with CPS < 1 as well [49].

## 4. What Comes Next in the Era of Immunotherapy?

**Figure 5:**
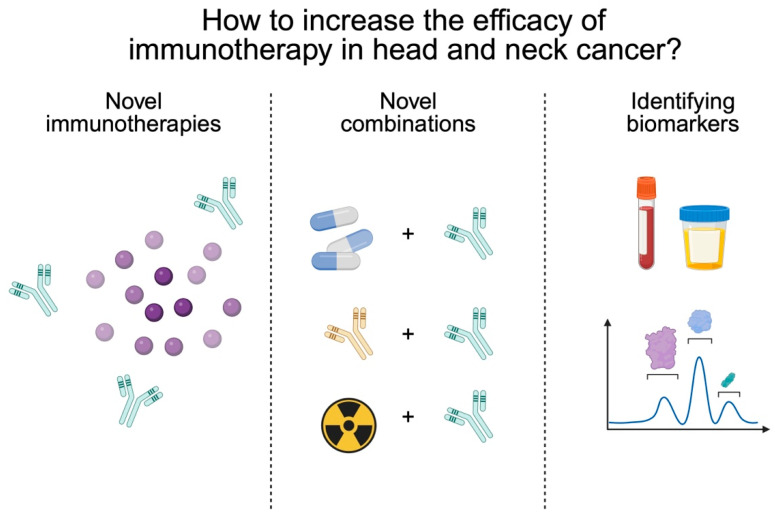
Research areas that aim to increase the effectiveness of immunotherapy in patients with head and neck cancer. Created in BioRender. Kiełbowski, K. (2026) https://BioRender.com/691jdip.

In the previous sections of this article, we have presented the latest results in the field of immunotherapy of patients with HNC. As demonstrated, numerous treatment agents and strategies have been examined, with more trials being currently performed. Some of these results are translated into clinical practice, like the use of immunotherapy in patients with recurrent/metastatic HNC. While we still expect the final results of several major clinical trials, researchers continue to study novel approaches to increase the efficacy and safety of immunotherapy. This is a matter of high importance, as the sustained response rate to this form of treatment remains unsatisfactory. It can be achieved by designing novel treatment agents or combinations and by identifying the most promising biomarkers of response, which will allow us to identify responders and non-responders more rapidly, thus changing treatment accordingly to limit exposure to ineffective therapies (Figure 5). Moreover, the efficacy of immunotherapy strongly relies on the composition of the TME, a highly complex structure composed of cancer cells, mesenchymal cells, immune cells, and molecules released by these cells. Together, the TME creates immunosuppressive conditions that prevent cytotoxic cellular activities. Several novel treatment approaches aim to modify properties of the TME.

Firstly, several authors have examined the potential use of new molecules and agents. To begin with, the clinical potential of targeting lymphocyte-activation gene 3 (LAG-3 or CD223) is being examined. Being an inhibitory molecule associated with T cell dysfunction, anti-LAG-3 inhibitors work similarly to other immunotherapeutics targeting PD-1/PD-L1 and CTLA-4. Due to the importance of suppressing LAG-3 and its clinical translation in oncology, the FDA approved an agent composed of relatimab (anti-LAG-3) and nivolumab in the treatment of melanoma [50]. In HNC animal models, a combination of anti-PD-1 and anti-LAG-3 therapeutics suppressed tumor growth and lymphatic metastasis. There were no significant differences between the combination strategy and anti-PD-1 group, but treatment composed of two immunotherapeutics achieved the largest significance compared to the control [51]. The use of an anti-LAG-3 drug (fianlimab) combined with cemiplimab (anti-PD-1) in humans with HNC was presented by Cho et al. [52] at the 2024 ASCO Meeting. The authors observed clinical activity and a manageable safety profile that requires further research. The results of several clinical trials investigating the efficacy and safety of anti-LAT-3 agents in HNC are expected (e.g., NCT04080804, NCT06494943, and NCT04811027).

IRX-2 is a multi-cytokine cell-derived agent that has been studied in neoadjuvant settings in patients with HNC. The treatment drug encompasses IL-1ß, IL-2, TNF-α, and IFN, crucial cytokines involved in immune responses. Perilymphatic administration was associated with significantly increased CD8+ cell infiltration, which was translated towards better prognosis [53]. Another approach was described by Talor et al. [54] in the IT-METTERS study, who examined the use of the leukocyte interleukin injection, which is a mixture of cytokines. The researchers analyzed the efficacy of this compound in neoadjuvant settings in patients with HNC. Trial design included three groups that received the leukocyte interleukins with or without cyclophosphamide, indomethacin, and zinc, as well as the standard of care group. The authors measured overall early response, determined 3 weeks after cytokine treatment. Forty-five patients achieved an early response, in whom significantly higher OS was noted, as compared to the groups of non-responders and standard of care. Interestingly, Yoon and collaborators [55] published a report of using neoadjuvant therapy in patients with early stage HNC. Specifically, the authors used a topical treatment with imiquimod, a toll-like receptor 7 (TLR)-7 agonist, which demonstrated activity in skin basal cell carcinoma [56,57]. In the study with HNC participants, imiquimod provided clinical regression in 14 out of the 15 analyzed patients. In addition, 60% of patients achieved more than 50% of tumor cell count reduction. Imiquimod is not an agent usually described as immunotherapeutic, but it has strong immunoregulatory properties. Specifically, researchers observed significant increases in the abundance of Tc and Th cells in tumor tissues. Perhaps, a combination of topical imiquimod with classic immunotherapy would provide further benefits in lesion regression.

Volrustomig is a PD-1/CTLA-4 bispecific monoclonal antibody that is currently being investigated in the eVOLVE-HNSCC phase III trial. The trial aims to determine the efficacy of the drug in the maintenance therapy of unresectable locally advanced HNC patients after concurrent radiochemotherapy [58]. The highly expected results will shed light on another potential use of immunotherapy in patients with HNC. Additionally, photoimmunotherapy is a relatively new and intriguing treatment approach. It involves the combination of monoclonal antibodies with light-reacting compounds and immunotherapy. ASP-1929 is an example of a combination of cetuximab with light-activatable dye. Red light activates the drug bound to the EGFR-expressing cells. This method kills cancer cells and synergizes with classic immunotherapy. In a phase Ib/II clinical trial investigating pembrolizumab with ASP-1929 in a cohort of patients with recurrent/ metastatic HNC, the ORR was 27.8%. Disease control rate (CR+PR+SD) was observed in 61.1% of patients [59].

Secondly, several novel treatment combinations were examined. Liu et al. [60] focused on studying senescence, a process associated with cell cycle arrest and aging. In the cohort of HNC patients treated with immunotherapy, the authors observed better responses in those with lower levels of immunosenescence. Subsequently, the researchers performed an in vivo experiment, studying the potential combination of immunotherapy with agents targeting senescence, known as senolytics. This approach was associated with reduced senescence and better outcomes in animals. The COIS-01 trial evaluated the safety and efficacy of tislelizumab combined with dasatinib and quercetin in 24 HNC patients, with 33.3% of participants achieving ORR. Only one of studied patients experienced a grade 3–4 treatment-related adverse event (TRAE), demonstrating the efficacy and safety of this combination. T cells in the TME are characterized by exhaustion, but they can still be divided into subcategories. Namely, T cells in the HNC can be extremely exhausted or they can maintain cytotoxic properties. It was suggested that patients likely to respond to immunotherapy have greater tumor infiltration of cells with functional cytotoxic properties. In an important publication by Oliveira et al., the researchers highlight that immunotherapy can reintroduce cytotoxic features of exhausted cells within the tumors, which creates the initial cellular population killing cancer cells. Importantly, initial surgical treatment removes all of these beneficial cells that could play an enormous role in first steps of anticancer treatment [61]. In an elegant review by Zhao et al. [62], the authors comprehensively discussed changes in intratumoral T cell populations induced by neoadjuvant immunotherapy. Briefly, differences within the profile of T cells can be observed between future responders and non-responders prior to treatment. Specifically, responders are associated with increased presence of resident memory-like CD8+ cells. After the introduction of treatment, a more pronounced decrease in activated Treg cells is observed in responders. With low rates of complete and major pathological responses, perhaps prolonged neoadjuvant immunotherapy would allow further advancements to be observed in intratumoral T cells.

Another study examined the use of immunotherapy with tadalafil, a phosphodiesterase-5 (PDE-5) inhibitor, after which it was demonstrated to have immunoregulatory and anticancer effects. Even though the combination treatment was not associated with significantly improved treatment response, tadalafil was found to alter immune genes. In HPV-positive patients, the drug promoted B-cell signatures while it enhanced B-cell and NK-cell gene expression in responders [63]. Thus, further research is indicated to identify a population that could achieve clinical benefits due to the immune-regulating features of tadalafil. Another molecule being explored in HNC is transforming growth factor-ß (TGF-ß). In HNC cells, TGF-ß induces cell cycle arrest, which is associated with epithelial-to-mesenchymal transition, cancer progression, and the development of metastasis [64]. Furthermore, the molecule is known to alter immunity within the TME, with recent studies examining the combination of immunotherapy with TGF-ß neutralization. The use of bintrafusp alfa, which targets PD-L1 and TGF-ß, induces profound immune-regulating effects, such as increasing the presence of circulating T cells, promoting the exhausted yet proliferating subset of T cells, and suppressing the Treg population [65]. Redman et al. [66] investigated the efficacy of bintrafusp alfa in 14 patients with HPV-unrelated HNC. In this cohort, five patients achieved PR, but CR was suspected in nodal disease. Exploratory analyses demonstrated that the beneficial role of the drug was unlikely linked to canonical signaling of TGF-ß. By contrast, influence on the immune system was suspected. The researchers noted increased infiltration of tumor immune cells but decreased presence of Tregs abundancy, which is in line with the previously mentioned study. In a cohort that received dual immunotherapy, depletion of effector Tregs was associated with a major histological response. Importantly, this change in Treg presence was linked with CD8+ cell transitions to effector and effector memory cells, highlighting a crucial role of Tregs in determining immunotherapy response [67]. Ficerafusp alfa is another bitherapeutic that targets TGF-ß with EGFR; it is being explored in HNC in combination with pembrolizumab [68]. The next recently investigated approach involves manipulating epigenetics. Mechanisms that control gene expression but do not directly affect DNA sequencing are known regulators of oncogenesis and treatment response. Qin et al. [69] studied the rechallenge of immunotherapy (durvalumab + tremelimumab) combined with 5-azacytidine, a DNA methyltransferase inhibitor. In a small-sample clinical trial, 58% of patients (7 out of 12) achieved OS longer than 12 months.

**Figure 6:**
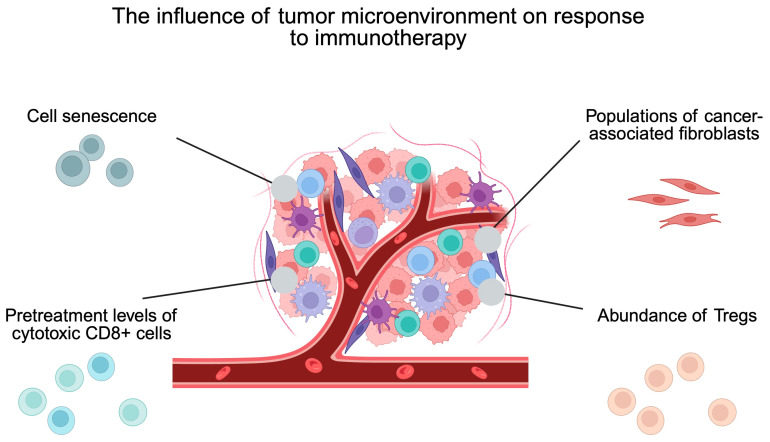
The influence of tumor microenvironment on response to immunotherapy. Created in BioRender. Kiełbowski, K. (2026) https://BioRender.com/yhnvdld.

Cancer-associated fibroblasts (CAFs) are other representatives of the TME cells. These cells are known to contribute to cancer growth through several mechanisms, including secretion of growth factors, promotion of angiogenesis, or shaping the extracellular matrix. Similarly to T cells, several categories of CAFs have been identified in HNC TMEs. Importantly, different phenotypes correlate with the presence of other cells, such as cytotoxic T cells [70]. These correlations could explain the relationships observed between different CAF populations and the response to immunotherapy [70,71]. Additionally, tumor cells within the TME can be studied to help in predicting the response to immunotherapy. Tumor-specific MHC-II was shown to differentially distinguish responders and non-responders to neoadjuvant immunotherapy. Interestingly, its biomarker potential was stronger than that of CPS [72]. Moreover, it was demonstrated that TME composition changes depending on the type of immunotherapy, demonstrating various pictures of response [73]. Therefore, identifying specific response reactions to individualized drugs could significantly affect the standard of care in patients treated with neoadjuvant immunotherapy. Accumulating evidence highlights the crucial role of the TME in shaping the response to immunotherapy (Figure 6). Therefore, an increasing number of studies examine methods to target the TME to improve antitumor responses and increase immunotherapy outcomes [74,75].

## 5. Multi-Omics, Spatial Dynamics, and TCR Repertoire Sequencing—How Can We Use Them Clinically?

**Figure 7:**
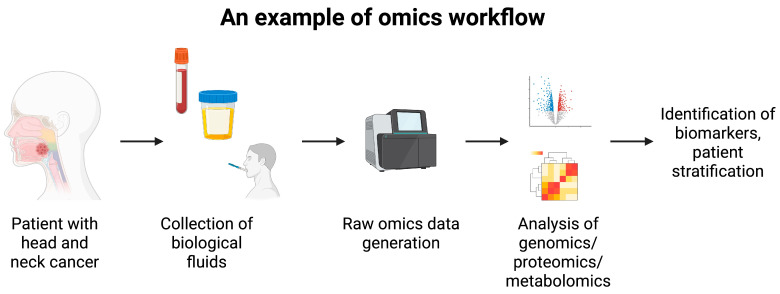
An example of omics workflow. Biological samples are collected and analyzed to obtain expression or abundance of hundreds of molecules. They are being processed to identify differentially expressed genes or differentially abundant molecules. Finally, the omics analyses aim to identify biomarkers and therapeutic targets, as well as to help stratify patients. Created in BioRender. Kiełbowski, K. (2026) https://BioRender.com/al99hff.

Omics is becoming one of the most popular types of research in oncology. Instead of classic analyses of single or several molecules, omics study whole biological systems, such as genes (genomics), transcripts (transcriptomics), and metabolism (metabolomics), among others. These analyses offer comprehensive insight into the biology of the studied samples. In oncology, omics can be used to identify biomarkers, stratify patients, or find therapeutic targets (Figure 7). In a recent report by Wu et al. [76], the authors utilized a multi-omics approach to analyze immunotherapy response in HNC patients. The authors identified a significant negative correlation between *A3A* and *EGFR* expression. Further analyses showed an extensive list of differentially expressed genes and differentially abundant proteins depending on *A3A* and *EGFR* expression. When comparing A3A-high, EGFR-low and A3A-low, EGFR-high cohorts, differently expressed RNA molecules were associated with 33 biological pathways. Within this group, 23 involved immune pathways. These findings corresponded to the analysis of immune-infiltrating cells. Specifically, A3A-high tumors showed enhanced presence of CD8+ cells and M1 macrophages, resembling an immunologically hot tumor environment. Subsequently, the authors confirmed the clinical utility of these observations, as patients with A3A-high expression tumors co-expressed PD-L1 and demonstrated better prognosis while treated with nivolumab [76]. This study further confirms the crucial role of immune pathways contributing to the response to immunotherapy. Additionally, it highlights the need for identifying molecules co-expressed with classic immune checkpoints. As demonstrated above, they could stratify patients into those likely to benefit from immunotherapy. Perhaps, the identification of groups of genes or proteins that associate or interact with immune checkpoints can lead to the formation of a biomarker panel that can be used clinically to select patients most likely to respond to particular agents. Furthermore, multi-omics approach offers the potential of selecting novel therapeutic targets. Given the previously mentioned example, we ask the following question: would targeting A3A provide benefit in patients resistant to nivolumab? Answering such questions will further drive the improvements in clinical outcomes. Another interesting approach was demonstrated by Sadeghirad and colleagues [77], who utilized proteomics, transcriptomics, and a digital spatial profiler to analyze immune cues in HNC tumor tissue. Researchers focused on tertiary lymphoid structures (TLSs) and germinal centers. Firstly, the authors demonstrated that HNC patients that respond to immunotherapy have higher expression of immunological proteins within TLSs, which is in line with other analyses concerning general tumor tissue and its influence on immunotherapy response. Intriguingly, spatial analysis demonstrated that greater distance between TLSs and tumor cells was associated with a poorer response to immunotherapy [77]. These findings provide us with a highly promising knowledge about the crucial role of tumor architecture. It adds another layer to the previously known impact of the composition of the TME and expression of immune-related molecules on responses to ICIs. The next promising area is T-cell receptor (TCR) repertoire sequencing or analysis. It studies the diversity of the TCR, which helps to understand the subtypes of present T cells and their protective functionalities [78]. Researchers have already suggested that studying TCR repertoires can be helpful in designing prognostic biomarkers, including in the cohorts treated with immunotherapy. In HNC, an interesting phenomenon can be observed. In an analysis of gamma delta TCR signatures, higher values were observed in HPV-positive tumors. Moreover, these signatures were associated with improved survival in this population, highlighting the influence of HPV on gamma delta T cells [79]. In addition, the clonality of T cells differs when tumor tissue, adjacent normal tissue, and lymphatic tissue are compared, which also demonstrates the specific change in T cell presence in HNC [80]. The TCR repertoire analysis was suggested to be used in the early detection of nasopharyngeal carcinoma [81], demonstrating one of the potential clinical benefits of this method. Overall, these modern and highly advanced tools offer great clinical translational potential. Apart from previously mentioned biomarker detection, patient stratification, and therapeutic target identification, they could help in qualification for neoadjuvant immunotherapy, hyper- or pseudoprogression prediction, and the identification of tumors requiring combinational approaches.

## 6. Conclusions and Future Perspectives

**Figure 8:**
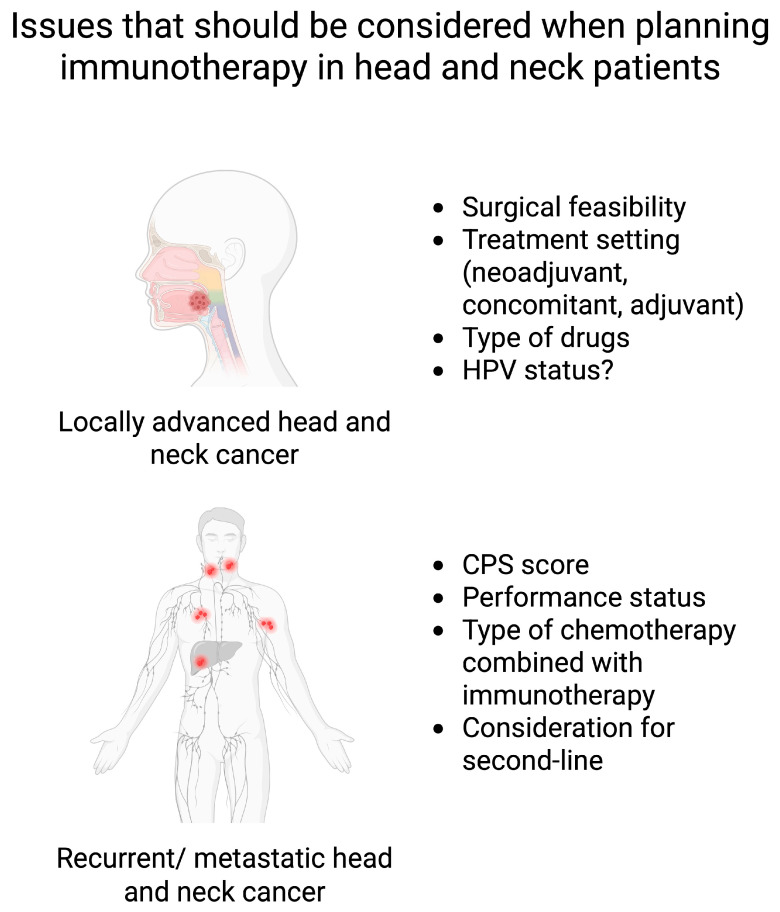
Integrating areas that need to be considered when planning immunotherapy in head and neck cancer patients. Created in BioRender. Kiełbowski, K. (2026) https://BioRender.com/8aegua9.

Immunotherapy has become a crucial element of modern oncology. It is used in clinical practice in a variety of malignancies. In HNC, many advancements in the field of immunotherapy have been made (Figure 8). For instance, the results of the KEYNOTE-048 trial led to the registration of pembrolizumab in patients with recurrent/metastatic HNC. Similarly, KEYNOTE-689 was a significant success for locally advanced resectable HNC. Furthermore, the role of nivolumab as a second-line treatment in HNC was established in the CheckMate-141 trial. The results of these studies induced a strong effect on treatment guidelines, with immunotherapy now being accepted and used as a treatment modality in HNC patients. The final results of other clinical trials, such as NIVOPOSTOP or CAMORAL, are greatly expected. The initial successes and promising results of other immunotherapy strategies and agents suggest potential novel opportunities in the near future. Nevertheless, the present review also aimed to demonstrate the difficult road for a new immunotherapy-based regimens to be approved. The negative results of the KEYNOTE-412, CompARE, or CheckMate 651 trials show that we need to look for cohorts most probable to benefit and drug combinations that will be associated with the greatest efficacy.

Apart from clinical trials, researchers are actively studying potential mechanisms that can improve the activity of immunotherapy. Over the years, we have gained significant knowledge about the crucial role of the TME in response to immunotherapy. With its complex structure involving different cell populations, their metabolism, and secreted molecules, it creates an immunosuppressive niche that strongly drives immunotherapy outcomes. Perhaps, this knowledge can translate into biomarker-driven highly precise therapeutic decisions that will improve the responses of oncological therapy. Administration of treatment guided by biomarkers should differentiate potential responders and non-responders, thus introducing adequate treatment methods and limiting exposure to non-effective therapies. These advances might be introduced with the use of omics, spatial dynamics or TCR repertoire analysis. With accumulating knowledge regarding the pathophysiology of HNC, there are still unexplored areas. For instance, the impact of HPV on the biology of cancer and its influence on treatment outcomes needs to be comprehensively examined. Recent studies demonstrated that HPV has a strong influence on immune responses in HNC [82].

## Figures and Tables

**Table 1:** A summary of FDA-approved immunotherapeutics.

Drug	Mechanism of Action	Indication	Reference
Pembrolizumab	Anti-PD-1 antibody	Head and neck squamous cell cancer Melanoma Non-small-cell lung cancer Malignant pleural mesothelioma Classical Hodgkin lymphoma Primary mediastinal large B cell lymphoma Urothelial cancer Colorectal cancer Gastric cancer Esophageal cancer Cervical cancer Hepatocellular cancer Biliary tract cancer Merkel cell carcinoma Renal cell carcinoma Endometrial carcinoma Cutaneous squamous cell carcinoma Triple-negative breast cancer	[1]
Nivolumab	Anti-PD-1 antibody	Head and neck squamous cell cancer Melanoma Non-small-cell lung cancer Malignant pleural mesothelioma Renal cell carcinoma Classical Hodgkin lymphoma Urothelial carcinoma Colorectal cancer Hepatocellular carcinoma Esophageal cancer Gastric Cancer, gastroesophageal junction cancer, and esophageal adenocarcinoma	[2]
Ipilimumab	Anti-CTLA-4 antibody	Melanoma Renal cell carcinoma Colorectal cancer Hepatocellular carcinoma Non-small-cell lung cancer Malignant pleural mesothelioma Esophageal cancer	[3]
Durvalumab	Anti-PD-L1 antibody	Non-small-cell lung cancer Small-cell lung cancer Biliary tract cancer Hepatocellular carcinoma Endometrial cancer Bladder cancer Gastric or gastroesophageal junction adenocarcinoma	[4]
Tremelimumab	Anti-CTLA-4 antibody	Hepatocellular carcinoma Non-small-cell lung cancer	[5]
Dostarlimab	Anti-PD-1 antibody	Endometrial carcinoma Mismatch repair deficient recurrent or advanced solid tumors	[6]
Cemiplimab	Anti-PD-1 antibody	Cutaneous squamous cell carcinoma Basal cell carcinoma Non-small-cell lung cancer	[7]
Atezolizumab	Anti-PD-L1 antibody	Non-small-cell lung cancer Small-cell lung cancer Hepatocellular carcinoma Melanoma Alveolar soft part sarcoma	[8]
Tislelizumab	Anti-PD-1 antibody	Esophageal cancer Gastric cancer	[9]
Avelumab	Anti-PD-L1 antibody	Merkel cell carcinoma Urothelial carcinoma Renal cell carcinoma	[10]
Cosibelimab	Anti-PD-L1 antibody	Cutaneous squamous cell carcinoma	[11]

## Data Availability

No new data were created or analyzed in this study. Data sharing is not applicable to this article.

## Abbreviations

The following abbreviations are used in this manuscript:

ICIs	Immune checkpoint inhibitors
PD-1	Programmed cell death protein 1
PD-L1	Programmed cell death protein ligand 1
CTLA-4	Cytotoxic T-lymphocyte-associated protein 4
HNC	Head and neck cancer
PFS	Progression-free survival
OS	Overall survival
CR	Complete response
PR	Partial response
SD	Stable disease
ORR	Overall response rate
5-FU	5-fluorouracil
CPXL	Carboplatin + paclitaxel
IDO1	Indoleamine 2,3 dioxygenase 1
TME	Tumor microenvironment
LAG-3	Lymphocyte activation gene 3
TLR	Toll-like receptor
TRAE	Treatment-related adverse event
PDE-5	Phosphodiesterase-5
TGF-ß	Transforming growth factor ß
CAFs	Cancer associated fibroblasts
TLS	Tertiary lymphoid structure
TCR	T-cell receptor
TIGIT	T-cell immunoreceptor with Ig and ITIM domains
LAG3	Lymphocyte activation gene 3
